# NSs Filament Formation Is Important but Not Sufficient for RVFV Virulence In Vivo

**DOI:** 10.3390/v11090834

**Published:** 2019-09-08

**Authors:** Shufen Li, Xiangtao Zhu, Zhenqiong Guan, Wenfeng Huang, Yulan Zhang, Jeroen Kortekaas, Pierre-Yves Lozach, Ke Peng

**Affiliations:** 1 State Key Laboratory of Virology, Wuhan Institute of Virology, Chinese Academy of Sciences, Wuhan 430071, China (S.L.) (X.Z.) (Z.G.) (W.H.) (Y.Z.); 2 Wuhan National Biosafety Laboratory, Mega-Science Center for Bio-Safety Research, CAS, Wuhan 430071, China; 3 University of Chinese Academy of Sciences, Beijing 100049, China; 4 Department of Virology, Wageningen Bioveterinary Research, 8211 Lelystad, The Netherlands; 5 Laboratory of Virology, Wageningen University, 6701 Wageningen, The Netherlands; 6 Cell Networks-Cluster of Excellence and Center for Integrative Infectious Disease Research, University Hospital Heidelberg, 69115 Heidelberg, Germany; 7 IVPC UMR754, INRA, Univ. Lyon, EPHE, 50 Av. Tony Garnier, 69007 Lyon, France

**Keywords:** RVFV, reverse genetics system, NSs, filament, virulence

## Abstract

Rift Valley fever virus (RVFV) is a mosquito-borne phlebovirus that represents as a serious health threat to both domestic animals and humans. The viral protein NSs is the key virulence factor of RVFV, and has been proposed that NSs nuclear filament formation is critical for its virulence. However, the detailed mechanisms are currently unclear. Here, we generated a T7 RNA polymerase-driven RVFV reverse genetics system based on a strain imported into China (BJ01). Several NSs mutations (T1, T3 and T4) were introduced into the system for investigating the correlation between NSs filament formation and virulence in vivo. The NSs T1 mutant showed distinct NSs filament in the nuclei of infected cells, the T3 mutant diffusively localized in the cytoplasm and the T4 mutant showed fragmented nuclear filament formation. Infection of BALB/c mice with these NSs mutant viruses revealed that the in vivo virulence was severely compromised for all three NSs mutants, including the T1 mutant. This suggests that NSs filament formation is not directly correlated with RVFV virulence in vivo. Results from this study not only shed new light on the virulence mechanism of RVFV NSs but also provided tools for future in-depth investigations of RVFV pathogenesis and anti-RVFV drug screening.

## 1. Introduction

Rift Valley fever (RVF) is a mosquito-borne anthropozoonosis caused by the RVF virus (RVFV), which belongs to the family *Phenuiviridae* in the order of *Bunyavirales* [1]. RVFV is a negative-sense single-stranded RNA virus with a segmented genome, comprising large (L), medium (M) and small (S) segments. The virus mainly circulates in a mosquito-ruminant transmission cycle, and other small mammals, such as rodents, may potentially participate in the maintenance of the virus [2]. Sheep are most susceptible to infection, manifesting with abortion of pregnant animals and acute mortality among newborns. Humans can become infected via contact with tissues and fluids released during the slaughtering of infected animals or via bites of infected mosquitoes [3]. Infected humans usually remain asymptomatic or develop a self-limiting febrile illness. However, in some cases, patients develop severe complications manifesting with acute hepatitis, encephalitis or hemorrhagic fever leading to death [4]. The demonstrated ability to cause large transboundary outbreaks explains the need for a safe and effective vaccine or antiviral therapy. Consequently, the World Health Organization has included RVF on the Blueprint list of priority diseases likely to cause future epidemics for which countermeasures are urgently needed (http://www.who.int/blueprint/priority-diseases/en/).

Since the virus was first isolated in Kenya in 1930 [5,6], subsequent outbreaks occurred in surrounding countries, after which the virus spread to South-, North- and West Africa and the Arabian Peninsula [7,8,9]. The first imported case of RVF in China was reported in 2016, when a Chinese worker returning from Angola was diagnosed with RVFV infection. The virus was isolated from the serum of the patient and was named the BJ01 strain [10]. Phylogenetic analysis revealed that the imported virus is a reassortant containing the L and M genes from lineage E and the S segment from lineage A [11]. This imported case underscores the risk for future emergence of RVFV in China and calls for preparedness programs.

The major virulence factor of RVFV is a non-structural 31-kDa protein named NSs that is encoded by the S segment. NSs localizes both in the cytoplasm and nucleus of infected cells, and it forms nuclear filamentous structures through homo-oligomerization [12]. NSs suppresses host innate immune responses through three strategies, including (i) inhibiting the type I interferon (IFN) system by stabilizing a repressor complex on the interferon-β (IFN-β) promoter [13]; (ii) dampening antiviral responses by targeting the RNA-dependent protein kinase (PKR) for degradation [14]; and (iii) inducing a host cellular transcription shut-off by disrupting the assembly of the RNA polymerase II transcription factor II H (TFIIH) complex [15]. Although various biological functions of NSs have been elucidated, it has remained unclear if NSs filament formation is a determinant of viral virulence. A recent study resolved the crystal structure of a truncated form of NSs (83–248 AA) of the attenuated MP-12 strain and, based on the structure, identified mutations in the fibril interfaces of NSs that abolish its nuclear filament formation (namly T1 and T3) [16]. However, the virulence of these NSs mutants with compromised NSs nuclear filament formation has not been investigated.

In this study, we generated a T7-based reverse genetics system to rescue the imported BJ01 strain. Both wild-type (WT) and mutant viruses lacking the NSs gene (r△NSs-eGFP) were recovered using this system. The replication properties and pathogenicity of the rescued viruses in vitro and in vivo were investigated. The recombinant BJ01 strain caused a severe cytopathic effect (CPE) in cell cultures and was highly pathogenic for BALB/c mice. To investigate the role of NSs filament formation in virulence, mutations previously shown to compromise NSs nuclear filament formation were introduced into the system. The phenotypes of the mutations were subsequently investigated in vitro and in vivo. The results showed that NSs filament formation is dispensible for efficient replication in vitro and appears important but insufficient for virulence in vivo.

## 2. Materials and Methods

### 2.1. Ethics Statement

Animal experiments were performed in agreement with Regulations for the Administration of Affairs Concerning Experimental Animals in China. The procedures were approved by the Laboratory Animal Care and Use Committee of the Wuhan Institute of Virology, Chinese Academy of Sciences (Wuhan, China) on 10 June 2019. The approval code was WIVA38201901.

### 2.2. Cells and Viruses

Baby hamster kidney (BHK-21), Huh7 and Vero cells were maintained in Dulbecco’s Modified Eagle Medium (DMEM; Gibco, Waltham, MA, USA) supplemented with 10% fetal bovine serum (FBS; Gibco) and 1% antibiotics (Gibco). BSR-T7/5 cells, stably expressing T7 RNA polymerase, were maintained DMEM supplemented with 10% fetal bovine serum and 1% antibiotics and additionally provided with G418. THP-1 cells were obtained from ATCC, and cultured in RPMI-1640 medium (Gibco) containing 10% FBS. All cell lines were grown in a humidified atmosphere of 5% CO_2_ at 37 °C. RVFV strains were propagated in BHK-21 cells under BSL-3 conditions, and viral titers were determined by plaque assay in Vero cells.

### 2.3. Plasmids

The pVSV-T7 plasmid, which contains a T7 promoter and terminator, was a gift from Prof. Gengfu Xiao at the Wuhan Institute of Virology. The cDNAs corresponding to the L, M and S segments of BJ01 were synthesized by reverse transcription-PCR (RT-PCR) from vRNA extracted from the supernatant of infected cells using a QIAamp viral RNA Mini Kit (Qiagen, Dusseldorf, Germany). The PCR products of antigenomes were cloned into pVSV-T7 between the T7 RNA polymerase promoter and terminator with the ClonExpress Ultra One Step Cloning Kit (Vazyme, Nanjing, China), resulting in T7 polymerase-driven plasmids (pT7-L, pT7-M and pT7-S). The NSs deletion and mutations in the oligomer interfaces (T1, T3 and T4) of NSs were constructed through homologous recombination using the plasmid pT7-S as a template.

### 2.4. Rescue of Recombinant Viruses

Recombinant WT (rWT) virus was rescued by transfecting monolayers of BSR-T7/5 cells (5 × 10^5^) with pT7-L, pT7-M and pT7-S, all of which were 1 μg with Lipofectamine 3000 (Life Technologies, Waltham, MA, USA). After 5 days, an obvious cytopathic effect was observed, and the supernatants were collected and passaged in BHK-21 cells.

The NSs deletion virus (r△NSs-eGFP) was recovered by co-transfecting BSR-T7/5 with pT7-L, pT7-M and pT7-S^△NSs-eGFP^ as described above.

The mutants (rT1, rT3 and rT4) were rescued by co-transfection with pT7-L, pT7-M and pT7-S with T1, T3 or T4 mutation in NSs.

### 2.5. Plaque Assay

Confluent Vero monolayers were infected with 10-fold dilutions of virus and incubated at 37 °C for 1 h. After absorption, culture medium was replaced by DMEM containing 2% FBS and 1.1% carboxymethyl-cellulose. After 7 days of incubation, the overlay was removed, and cells were stained with 1% crystal violet after fixing with 3.6% formaldehyde.

### 2.6. Western Blot Analysis

BHK-21 and Huh7 cells infected with indicated viruses were lysed with buffer (50 mM Tris, pH 7.5, 150 mM NaCl, 1% NP40, 5 mM EDTA and 10% glycerol). Cell lysates were subjected to 12% SDS-polyacrylamide gel electrophoresis (PAGE) then transferred to polyvinylidene difluoride (PVDF) membranes (Millipore, Darmstadt, Germany). Proteins were incubated with primary antibodies against nucleocapsid protein (NP), NSs and Tubulin (Abclonal, Wuhan, China), then secondary horseradish peroxidase-conjugated goat anti-rabbit IgG. Protein bands were detected by an enhanced chemiluminescence (ECL) kit (Millipore) using a Chemiluminescence Analyzer (Chemiscope600pro).

### 2.7. Electron Microscopy (EM) of Infected Cells

Huh7 cells (2 × 10^6^) were infected with RVFV WT (BJ01), rWT or rescued virus lacking the NSs gene (r△NSs-eGFP) at an MOI of 1 for 24 h. Cells were washed three times with PBS, fixed with 2.5% (*w*/*v*) glutaraldehyde in 0.1 M sodium chloride and and processed for Electron Microscopy (EM). The viral particles were observed under a transmission electron microscope (FEI Tecnai G2 microscope at 200 kV).

### 2.8. Fluorescence and Immunofluorescence Microscopy

BHK-21 cells infected with r△NSs-eGFP were fixed with 4% paraformaldehyde (PFA) and incubated with 4′,6-diamidino-2-phenylindole (DAPI). BHK-21 cells infected with rWT were fixed and permeabilized with 0.2% (vol/vol) Triton X-100, then blocked with PBS containing 3% bovine serum albumin (BSA). Cells were incubated with anti-NP antibody for 1 h at room temperature (RT), followed by incubation with Alexa Fluor 488-conjugated goat anti-rabbit IgG, then washed and incubated with DAPI. Images were captured by an epifluorescence microscope (Olympus IX73).

For the immunostaining of NSs, Vero and Huh7 cells were mock treated or infected with rWT and mutants (rT1, rT3 and rT4) at an MOI of 5. At 24 h p.i., cells were fixed and permeabilized as described above, then blocked with 3% BSA. Cells were incubated with anti-NSs antibody for 1 h at RT, followed by incubation with Alexa Fluor 561-conjugated goat anti-rabbit IgG. After that, cells were incubated with DAPI, and analyzed using a confocal microscope (Andor Dragonfly 202).

### 2.9. Determination of In Vivo Virulence of Mutant Viruses

BALB/c mice were purchased from Charles River Laboratories (Beijing, China). Sixty 6-week-old WT BALB/c mice were divided into six groups (*n* = 10). Five groups of mice were intraperitoneally injected with 5 plaque forming unit (PFU) of rWT, rT1, rT3, rT4 or r△NSs-eGFP in 100 μL of PBS. As a control, a group of mice were injected with PBS. Mice were monitored for clinical signs and weighed once a day, then humanely euthanized at day 5 or 7. Liver and spleen samples were harvested for analysis. RNA viral loads in liver and spleen were determined by quantitative RT-PCR.

### 2.10. Quantitative RT-PCR

RNA was extracted from the liver and spleen homogenates using TRIzol Reagent (Life technology, Waltham, MA, USA) according to the manufacturer’s instructions. Quantitative RT-PCR was performed using the HiScript II One Step qRT-PCR SYBR Green Kit (Vazyme, Nanjing, China) and the Bio-Rad CFX96 Real-Time System. Standard curves were drawn using a RVFV RNA positive control obtained through in vitro synthesis.

### 2.11. Statistical Analyses

Statistical analyses were performed in GraphPad Prism version 6, as defined in the text and figure legends. A *p* value < 0.05 was considered to be statistically significant.

## 3. Results

### 3.1. Rescue of RVFV BJ01 and a Mutant Lacking the NSs Gene

**Figure 1:**
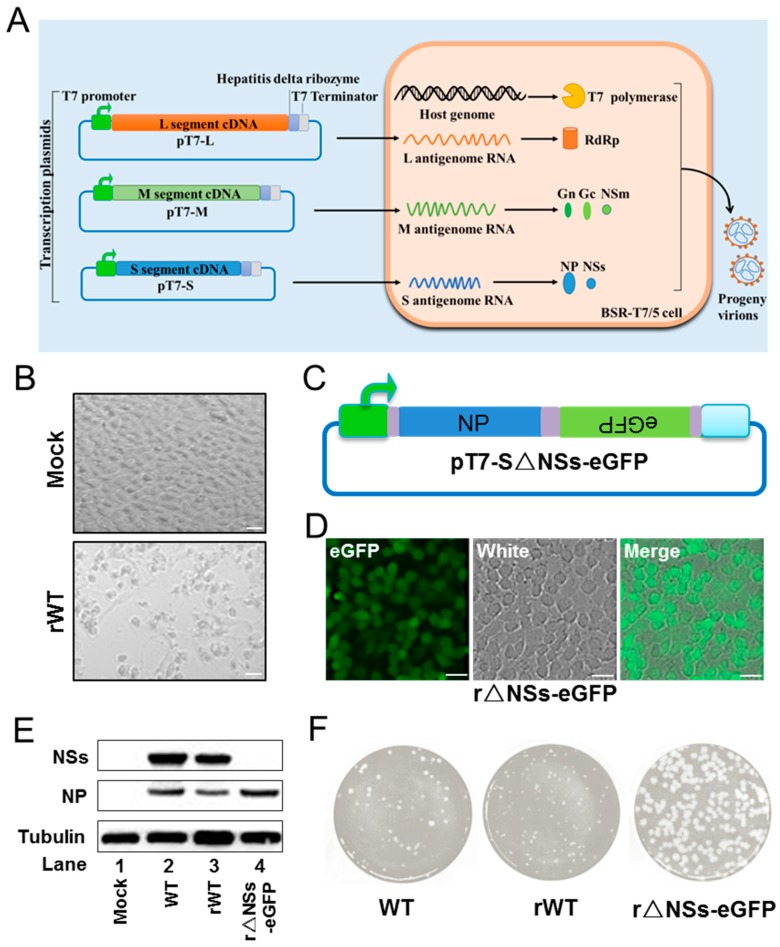
Rescue of RVFV BJ01 and a mutant lacking the NSs gene. (**A**) Schematic of the T7 RNA polymerase-dependent reverse genetics system for construction of recombinant viruses Adapted from the Box 2 figure of Orthobunyaviruses: recent genetic and structural insights [18]. (**B**) Cytopathic effect (CPE) of rescued virus in BHK-21 cells. BHK-21 cells were mock treated or infected with rWT at an MOI of 5, images were captured at 48 h p.i. Bars, 20 μm. (**C**) Construction of the plasmid encoding the S segment in which the NSs gene was replaced by eGFP. (**D**) Fluorescence microscopy analysis to verify the successful rescue of r△NSs-eGFP. BHK-21 cells were infected with r△NSs-eGFP (MOI = 5) and the fluorescence images were acquired at 72 h p.i. Bars, 20 μm. (**E**) Western blot analysis of infected cells with anti-N, anti-NSs and anti-Tubulin antibodies. Vero cells were infected with WT, rWT and r△NSs-eGFP at an MOI of 1 and cell lysates were harvested at 24 h p.i. (**F**) Plaque assay of RVFV WT and rescued viruses (rWT and r△NSs-eGFP) on Vero cells. After 7 days of incubation, plaques were stained with crystal violet.

The RVFV BJ01 strain (GenBank accession numbers No. KX632066, KX632067 and KX632068, respectively) was isolated from the patient of the first RVF case imported into China [10]. Using a T7 RNA polymerase-dependent system that relies on the T7 promoter for the synthesis of viral transcripts, the anti-genomic full-length segments L, M and S from RVFV were cloned into the vector pVSV-T7, which contains a T7 promoter and terminator. The resulting plasmids, named pT7-L, pT7-M and pT7-S, were transfected into BSR-T7/5 cells. The system is depicted in Figure 1A. Five days post transfection, CPE was observed in cells transfected for viral rescue but not in mock transfected cells (Figure 1B). Rescue of viruses from plasmids was confirmed by an intentionally introduced mutation in the S segment. Briefly, viral RNA was extracted from supernatants of cells infected with the wild-type (WT) virus and from those infected with the recombinant derivative (rWT). The sequence containing the mutation was amplified by RT-PCR and subjected to sequencing. Sequencing detected the intentionally introduced mutation, confirming that rWT was derived from plasmids (Figure S1). In addition, we recovered a recombinant virus lacking the NSs gene, in which the entire NSs coding sequence was replaced with the eGFP gene (Figure 1C). The resulting virus, named r△NSs-eGFP, was shown to express eGFP in infected BHK-21 cells without causing strong CPE (Figure 1D). Western blot analysis with respective antibodies showed that the expression of N protein was detected in WT, rWT and r△NSs-eGFP-infected cells, and that NSs was absent in r△NSs-eGFP-infected cells, confirming the successful deletion of NSs (Figure 1E). Plaque assays revealed that while WT and rWT viruses showed typical plaque with a clear boundary, the r△NSs-eGFP infection resulted in larger plaques, as described previously (Figure 1F) [17]. Taken together, these results suggest that the BJ01 strain was successfully rescued using the T7 RNA polymerase-dependent system.

### 3.2. Characterization of Rescued Recombinant Viruses

**Figure 2:**
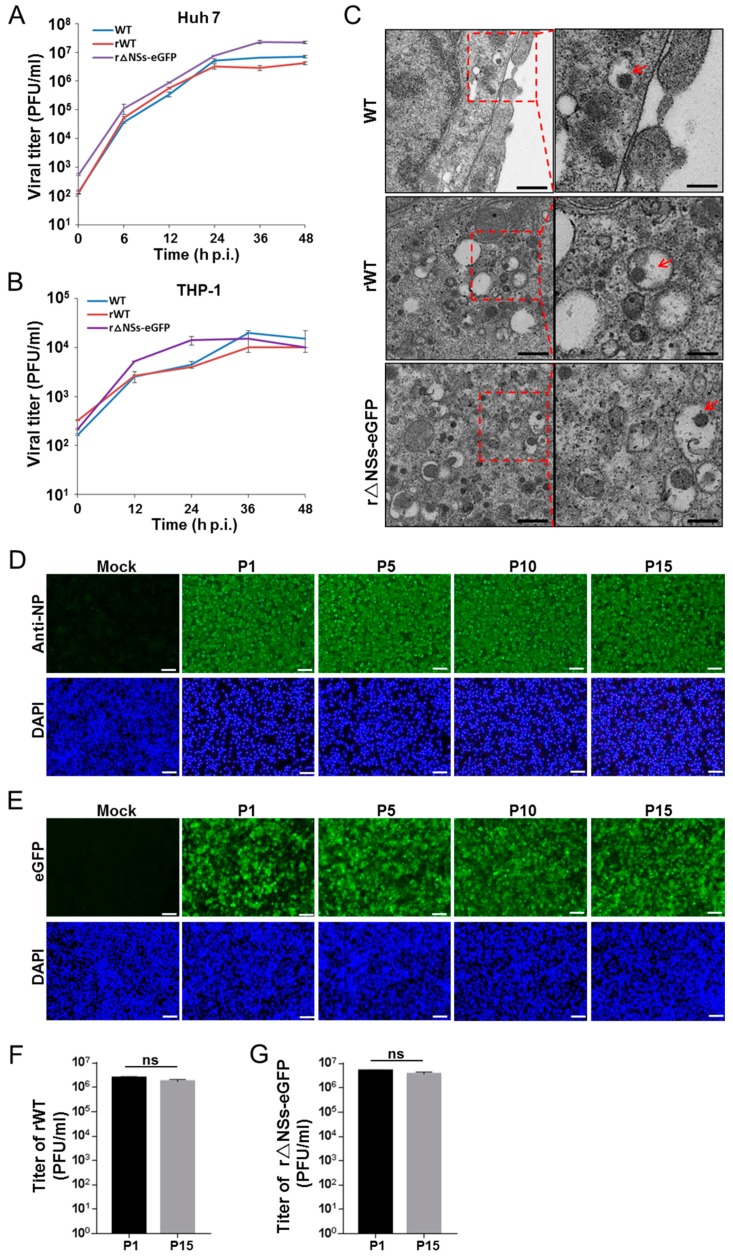
Characterization of rescued recombinant viruses. (**A**,**B**) One-step growth kinetics of WT, rWT and r△NSs-eGFP in Huh7 (**A**) and THP-1 cells (**B**). Cells were infected with a 0.1 MOI for Huh7 or 1 MOI for THP-1, and supernatants were collected at indicated time points. Viral titers were detected by plaque assay on Vero cells; error bars represent standard deviation. (**C**) EM analysis of progeny virus formation. Huh7 cells were infected with WT, rWT or r△NSs-eGFP. At 24 h p.i., cells were fixed and observed by TEM. The images show that virus particles were located in the cytoplasm of infected cells. Right panels are enlarged pictures of the boxed regions, red arrows indicate virus particles. Bars are 1 μm (left panels) and 500 nm (right panels). (**D**,**E**) Immunofluorescence and fluorescence microscopy to detect rWT (**D**) and r△NSs-eGFP (**E**) infection in BHK-21 cells from each passage. The images were taken from different passages. Green fluorescence indicates infection. P1 represents the first passage; P5, the fifth passage; P10, the tenth passage; and P15, the fifteenth passage. Bars, 50 μm. (**F**,**G**) The virus titer of rWT (**F**) and r△NSs-eGFP (**G**) from indicated passage numbers in BHK-21 cells was detected by plaque assay. P1, the first passage; P15, the fifteenth passage.

In order to characterize the recombinant RVF viruses rescued from plasmids, one-step growth kinetics of WT, rWT and r△NSs-eGFP in Huh7 cells and THP-1 cells were performed. The results show that the replication kinetics of WT, rWT and r△NSs-eGFP were similar in both cell lines (Figure 2A,B), indicating that replacing the NSs ORF with the sequence-encoding eGFP did not strongly affect virus growth in these cell lines. Infection of Huh7 or THP-1 cells with WT or rWT viruses resulted in clear CPE, but no CPE was observed in r△NSs-eGFP virus-infected cells, as observed in Vero and BSR-T7/5 cells. The viruses were found to amplify more efficiently in Huh7 cells, since the titers of viruses in the supernatant of infected Huh7 cells were higher than those obtained from infection of THP-1 cells (Figure 2A,B). The progeny virus formation was further analyzed by electron microscopy (EM) and virus particles with a diameter of ~80 to 120 nm were observed in the cytoplasm of infected cells (Figure 2C), which is in agreement with previous work [19]. To analyze the genetic stability of rescued RVFV viruses, rWT and r△NSs-eGFP were serially passaged on BHK-21 cells. Supernatants containing the viruses of passage 1, 5, 10 and 15 were collected and the infectivity was monitored either by immunofluorescence with anti-N antibody or by detecting eGFP signal in the infected cells. Similar infectivity was observed for both viruses from these passages (Figure 2D,E). Furthermore, plaque assay showed that the virus titers were comparable between the 1st and 15th passages for both viruses (Figure 2F,G). These results together suggest that rWT and r△NSs-eGFP were genetically stable during in vitro passages.

### 3.3. Rescue of Recombinant RVFVs with Mutations Affecting NSs Filament Formation

**Figure 3:**
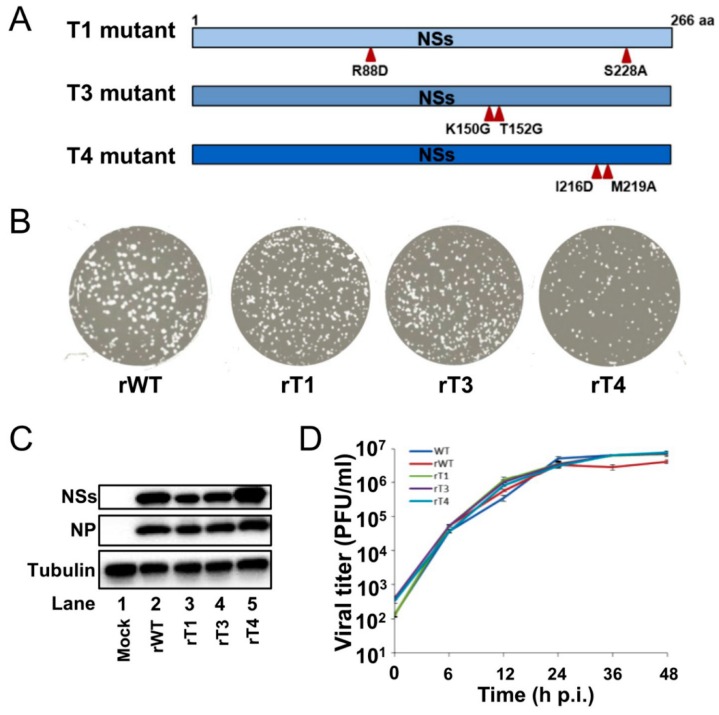
Rescue of recombinant RVFVs with mutations that affect the NSs filament formation. (**A**) Schematic representations of NSs mutations introduced into the reverse genetics system. (**B**) Plaque assay of rWT and mutant viruses on Vero cells. Plaques were stained with crystal violet at 7 days p.i. (**C**) Western blot analysis of infected cells. Huh7 cells were infected with rWT and mutant viruses at an MOI of 1, and cell lysates were harvested and analyzed at 24 h p.i. with indicated antibodies. (**D**) Replication kinetics of WT, rWT and mutant viruses in Huh7 cells. Huh7 cells were infected with respective viruses with an MOI of 0.1 and the supernatants were collected at 0, 6, 12, 24, 36 and 48 h p.i. Viral titers were detected by plaque assay on Vero cells; error bars represent standard deviation.

NSs is reported to be the major virulence factor of RVFV and forms unique filaments in the nucleus of an infected cell. A correlation between NSs filament formation and virulence has been proposed but not been extensively investigated in vivo. A previous study reported mutations in the interface of NSs oligomers that might abrogate its nuclear filament formation (namely T1 (R88D, S228A) and T3 (K150G, T152G), while T4 (I216D, M219A) still forms nuclear filament) [16]. This set of mutations therefore provides ideal tools to analyze the potential correlation between NSs filament formation and virulence. For this purpose, the reported mutations in the interface of NSs (T1, T3 and T4) were introduced into the NSs gene of the reverse genetics system (Figure 3A). Recombinant viruses were recovered (namely, rT1, rT3 and rT4) and the mutations were confirmed by sequencing of the S segment (Figure S2). Viral progeny was detected by plaque assay, revealing similar plaque morphologies of rWT and mutant viruses (Figure 3B). Western blot analysis of infected cells confirmed the expression of both N protein and NSs by the mutant and WT viruses in infected Huh7 cells (Figure 3C). In order to assess whether NSs mutations affect virus replication kinetics, Huh 7 cells were infected with rWT or mutants with an MOI of 1, and the virus titers were determined by plaque assay at different time points post infection. All four viruses showed growth kinetics similar to that of the parental WT virus in Huh7 cells, indicating that these NSs mutations did not affect virus replication in vitro (Figure 3D).

### 3.4. Analysis of NSs Filament Formation in Infected Cells

**Figure 4:**
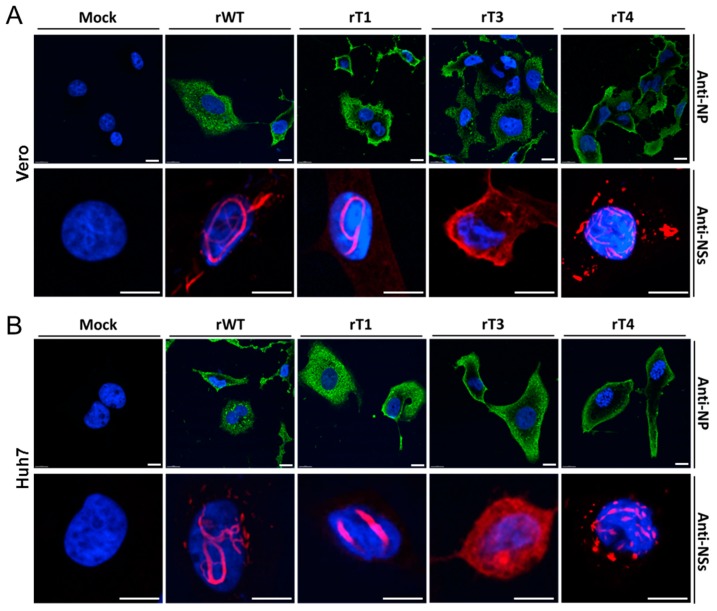
Analysis of NSs filament formation in infected cells. Vero (**A**) and Huh7 (**B**) cells were infected with indicated viruses (MOI = 5) and fixed at 24 h p.i. The infection and filament formation were analyzed by immunofluorescence microscopy using anti-N (green) or anti-NSs antibodies (red) as indicated. Nuclei were stained with DAPI. Bars, 10 μm.

Next, the impact of these mutations on NSs filament formation was analyzed in infected cells. For this purpose, Vero or Huh7 cells were infected with rWT, rT1, rT3 and rT4 with an MOI of 5 for 24 h. Afterwards, cells were fixed and probed with antibodies against N or NSs proteins followed by immunofluorescence microscopy analysis. As shown in Figure 4A, in rWT-infected Vero cells, NSs formed distinct filaments in the nucleus of infected cells and also localized in the cytoplasm, as previously reported [20]. Unexpectedly, typical filament formation was also observed in the nucleus of cells infected with rT1, which is different from the previous study in which nuclear aggregation of NSs but no filament formation was observed for this mutation [16]. Also different from the previous study, NSs of the rT3 mutant was observed nearly entirely in the cytoplasm in this study, displaying a diffusive distribution, while it was previously reported that T3 NSs was largely localized in the nucleus [16]. NSs filament formation was observed in the nucleus of rT4-infected cells and, unlike the typical filament structure, the rT4 NSs filaments appeared in shorter and fragmented forms (Figure 4A). Similar results were also observed in virus-infected Huh7 cells, indicating that these phenotypes are not cell-type specific (Figure 4B). Taken together, these results showed that these NSs mutants displayed different intracellular localization and patterns, and could be employed for in vivo evaluation of the correlation between NSs filament formation and virulence.

### 3.5. The NSs Mutant Viruses Showed Compromised Virulence In Vivo

These NSs mutant viruses were then employed to investigate the potential correlation between NSs filament formation and in vivo virulence. First, the LD_50_ was determined for rWT in BALB/c mice. Mice were infected with rWT RVFV through intraperitoneal injection with different amounts of viruses ranging from 1 to 100 PFU, and infected mice were monitored on a daily basis. The BALB/c mice were highly susceptible to the rescued rWT RVFV and began to die 3 days post infection (Figure S3A). The LD_50_ for this strain was determined to be 2.15 PFU, indicating robust virus replication and a potent virulence effect. No significant change in body weight was observed except for the high dose group, for which 100 PFU of virus was injected (Figure S3B). The virus demonstrated a broad tropism and could be detected in the liver, spleen, kidney and brain. The virus titer was the highest in the liver, indicating the liver as a main location for viral replication, similar with other virulent strains reported previously (Figure S3C) [21].

**Figure 5:**
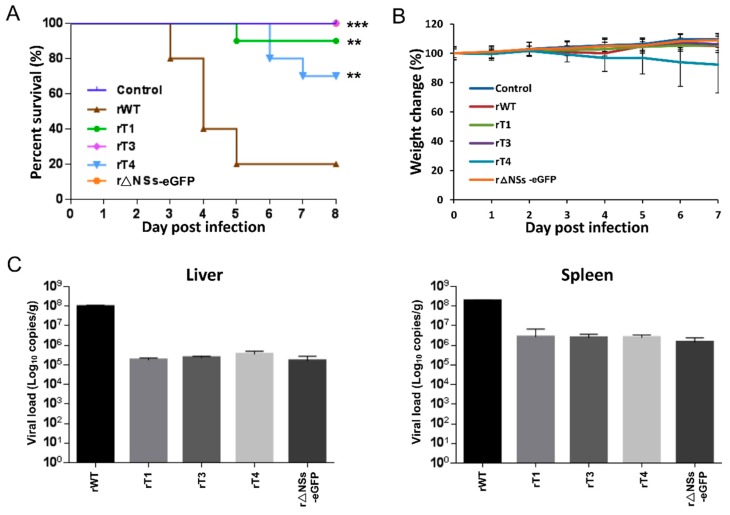
Analysis of the in vivo virulence of NSs mutant viruses. Six-week-old BALB/c mice were intraperitoneally inoculated with 5 PFU of rWT, rT1, rT3, rT4 or r△NSs-eGFP, or inoculated with PBS as a control. The mice in each group (*n* = 10) were observed for 7 days on a daily basis after infection. The mice survival rates (**A**) and body weights (**B**) were evaluated. Error bars represent standard deviation. Comparison of survival curves between mutants and rWT was analyzed by Log-rank (Mantel–Cox) testing. *** *p* < 0.001, ** *p* < 0.01. The *p* values were calculated based on comparison with the rWT. (**C**) Liver and spleen samples were harvested from infected mice 5 days post infection. RNA was extracted from tissue homogenates using TRIzol Reagent. RNA viral load in liver and spleen were determined by quantitative RT-PCR.

In order to evaluate the potential correlation between NSs filament formation and virulence, BALB/c mice were intraperitoneally injected with rWT, rT1, rT3, rT4 and r△NSs-eGFP viruses and monitored daily. Ten mice were infected for each group (*n* = 10) and infection was performed with 5 PFU with the purpose of achieving a higher fatality rate. As a control, a group of mice were injected with PBS. As shown in Figure 5A, while the rWT virus resulted in a fatality rate of 80%, the rT4 and rT1 displayed a strongly reduced virulence, resulting in fatality rates of 30% and 10%, respectively. Infection with rT3 or r△NSs-eGFP viruses did not result in any fatal infection and no clinical signs were observed for these viruses as with the PBS control (Figure 5A). Of these infections, only rT4 infection led to significant weight loss (over 25% in 3 out of 10 mice) (Figure 5B). When the RNA viral loads were determined from the liver and spleen from infected mice, it was found that the RNA viral loads of NSs mutant viruses and the r△NSs-eGFP virus were much lower compared to the rWT virus (Figure 5C). This reduced replication capacity is consistent with the compromised virulence.

## 4. Discussion

Investigating the virulence mechanism of RVFV is critical for understanding its pathogenesis, and NSs was identified to be the key virulence factor in vivo. NSs forms a distinct filament structure in the nuclei of infected cells and it has been proposed that the filament structure formation is associated with its virulence, although the mechanism still awaits further characterization [22]. Recently, the crystal structure of a truncated NSs protein (83-248 AA) was determined and several mutations on NSs were shown to be detrimental to NSs filament formation (namely T1 (R88D, S228A) and T3 (K150G, T152G)). For this reason, we introduced these mutations into NSs in our system. The NSs T4 mutant (I216D, M219A) was reported to still form nuclear filament and was included in parallel as a control. These mutant viruses showed comparable replication kinetics as theWT virus in vitro. Unexpectedly, we found that the NSs T1 mutant still formed distinct nuclear filament structure in both Vero and Huh7 cells, indicating that this mutation did not affect NSs filament formation in our system. Also different from the previous report, we observed that the NSs T3 mutant displayed a diffusive distribution in the cytoplasm of infected cells, while it was reported to form aggregates in the nuclei of infected cells. The NSs T4 mutant formed nuclear filaments but, unlike the distinct filament structure of WT NSs, the NSs T4 filament appeared as fragmented structures. Currently the reasons for these apparent inconsistencies are unclear; however, it is noteworthy that a virulent wild-type strain was used in this work, whereas the study of Barski and co-workers made use of the attenuated MP-12 strain [16], which the NSs protein differs from by five amino-acids from the BJ01 strain (Figure S4). Compared to the other field isolate of RVFV strains, ZH548 and ZH501, the NSs of BJ01 contains four amino-acid substitutions (Figure S4). Whether these differences in amino acid sequence may lead to different NSs filament formation patterns would be an interesting question for future investigation.

The three NSs mutants showed different intracellular patterns, presenting a unique opportunity to investigate the correlation between NSs filament formation and its virulence. Notably, although the T1 mutant still formed nuclear filaments in infected cells, the virulence of this mutant was strongly reduced in comparison with the WT virus (10% vs. 80%). Similar results were also obtained with the T4 mutant, for which a 30% fatality rate was recorded. On the other hand, the T3 mutant, which was impaired in NSs filament formation, did not cause lethal infection in mice, similar to the r△NSs-eGFP virus. These findings suggest that NSs filament formation is important per se, but probably not sufficient for its in vivo virulence. It remains intriguing how the mutations in T1 and T4 affect virulence. Notably, although replication of the T1 and T4 mutants were comparable with the WT virus in cell culture, replication of these two mutant viruses was significantly reduced in vivo, which may be related to the reduced virulence. Nevertheless, these mutant NSs viruses can be employed for future in-depth investigation of NSs virulence mechanisms.

No nuclear localization signal (NLS) has been identified for NSs so far, and it was proposed that interaction with host factors might mediate nuclear localization [23]. Interestingly, the NSs T3 mutant showed a cytoplasmic diffusive distribution, indicating that the amino acids K150 and T152 might be critical for mediating NSs nuclear translocation. Comparing the interacting host factors of the WT and T3 NSs might allow for the identification of relevant NSs–host interactions that mediate NSs nuclear translocation. On the one hand, NSs has a molecular weight of 31 kDa, and should be able to freely translocate through the nuclear pore complex [24]. On the other hand, the T3 mutant was observed nearly entirely in the cytoplasm, indicating that it might form a protein complex with itself and/or with host factors, leading to its sequestration in the cytoplasm. The diffusive distribution of the T3 mutant also indicates that the amino acids K150 and/or T152 might be key amino acids that determine NSs homo-oligomerization and filament formation.

Results from this study also lay the basis for several potential applications. The NSs mutants with compromised virulence might find application in vaccine development to further improve the live attenuated RVFV vaccine candidates. Whether combinations of these NSs mutations would further increase safety and reduce the chance of occurrence of revertant mutations would be interesting questions for future investigation. Additionally, the reporter gene of eGFP was successfully inserted into the reverse genetic system through replacing the NSs coding sequence. This recombinant virus showed robust genetic stability and the reporter gene was efficiently expressed even when the virus had been serially passaged for 15 passages. This recombinant reporter virus has the potential to be used for future anti-RVFV drug screening. These efforts would contribute to the development of effective countermeasures (anti-viral drugs and vaccines) to contain the threat of this highly pathogenic virus from both domesticated animals and humans.

## Supplementary Materials

The following are available online at https://www.mdpi.com/1999-4915/11/9/834/s1, Figure S1. Sequencing verification of virus rescue; Figure S2. Sequencing verification of recombinant RVFVs; Figure S3. Determination of LD_50_ for the rWT virus; Figure S4. Alignment of NSs amino acid sequences of BJ01 and MP12.
