# Supplementary material for: NSs Filament Formation Is Important but Not Sufficient for RVFV Virulence In Vivo
Source: Viruses. 2019 Sep 8;11(9):834. doi: 10.3390/v11090834 (PMC6783917; doi:10.3390/v11090834)
Supplement: Supplementary file 1 [file viruses-11-00834-s001.pdf]

Supplementary Materials

Materials and Methods

Viral RNA extraction

BHK-21 cells were infected with indicated viruses at an MOI of 5. The supernatants were collected at 48 h p.i., and viral RNA was extracted using the QIAamp viral RNA Mini Kit (Qiagen, Germany) according to the manufacturer's instructions.

Determination of LD_{50}

BALB/c mice were purchased from Charles River Laboratories (Beijing, China). Twenty 6-week-old WT BALB/c mice were divided into four groups (n=5). Three groups of mice were intraperitoneally injected with 10-fold dilutions of rWT in 100 μl of PBS. As control, a group of mice were injected with PBS. Mice were monitored for clinical signs and weighed once a day, and humanely euthanized at day 5. Brain, kidney, liver and spleen samples were harvested. RNA viral load in tissue samples were determined by quantitative RT-PCR.

Quantitative RT-PCR

RNA was extracted from the liver and spleen homogenates by using Trizol Reagent (Life technology, USA) according to the manufacturer's instructions. Quantitative RT-PCR was performed using the HiScript II One Step qRT-PCR SYBR Green Kit (Vazyme, China) and the Bio-Rad CFX96 Real-Time System. Standard curves were drawn using a RVFV RNA positive control obtained by *in vitro* synthesis.

Fig. S1.

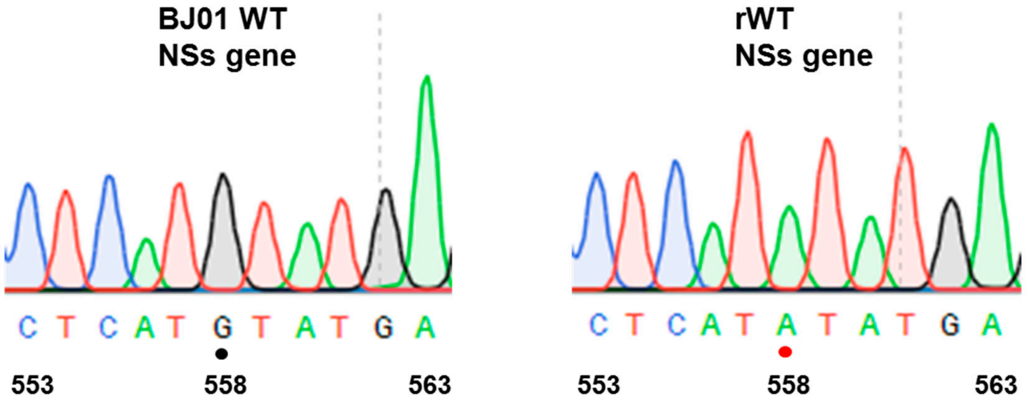

Figure. S1. Sequencing verification of virus rescue. Sequence analysis of the NSs gene of rescued RVFV WT compared to that of BJ01 carried out by RT-PCR of viral RNA purified from supernatant of infected BHK-21cells. The dot indicates mutation site.

Fig. S2.

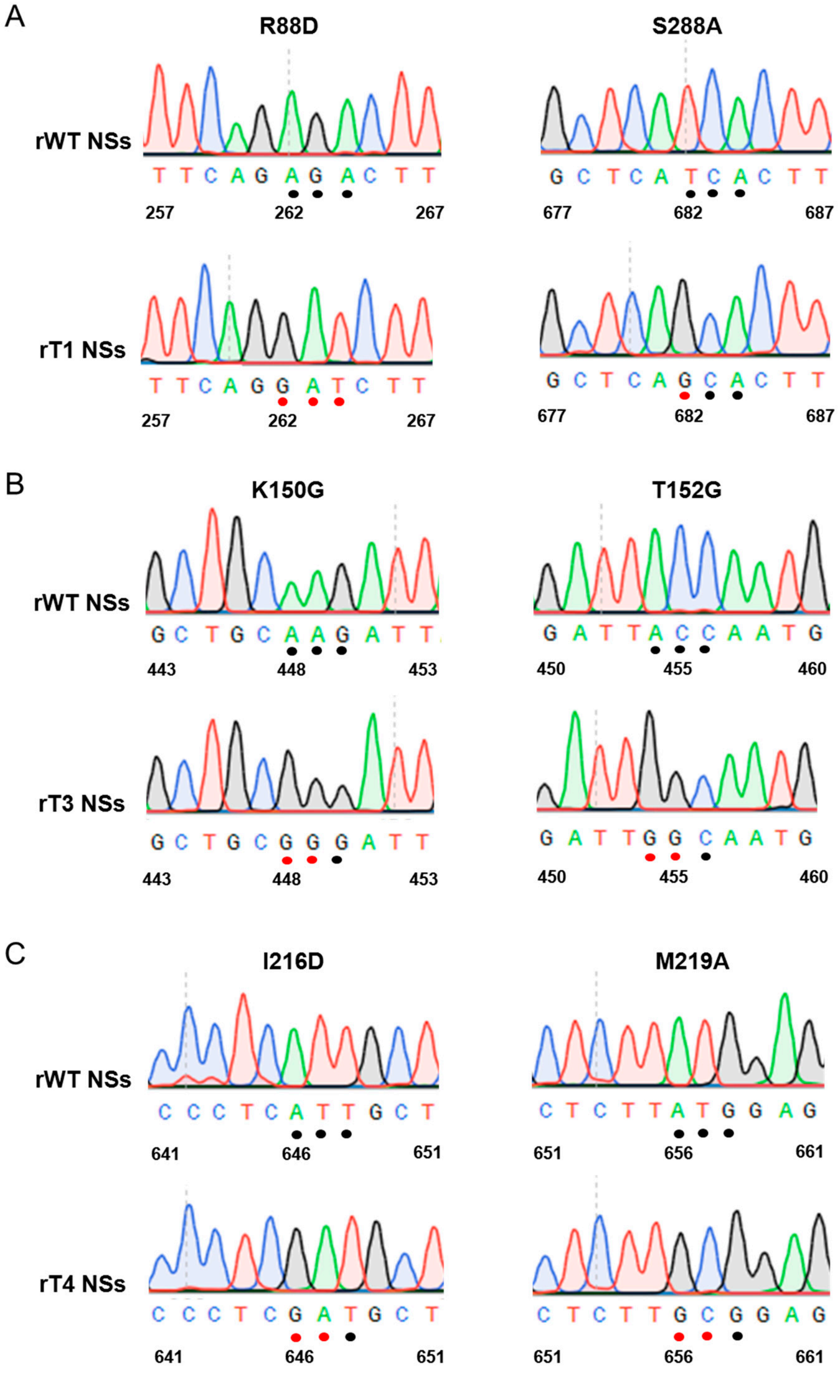

Figure. S2. Sequencing verification of recombinant RVFVs. Sequence analysis of the NSs gene of rT1 (A), rT3 (B) and rT4 (C) compared to that of rWT carried out by RT-PCR of viral RNA purified from supernatant of infected BHK-21cells. The dots indicate mutation sites.

Fig. S3.

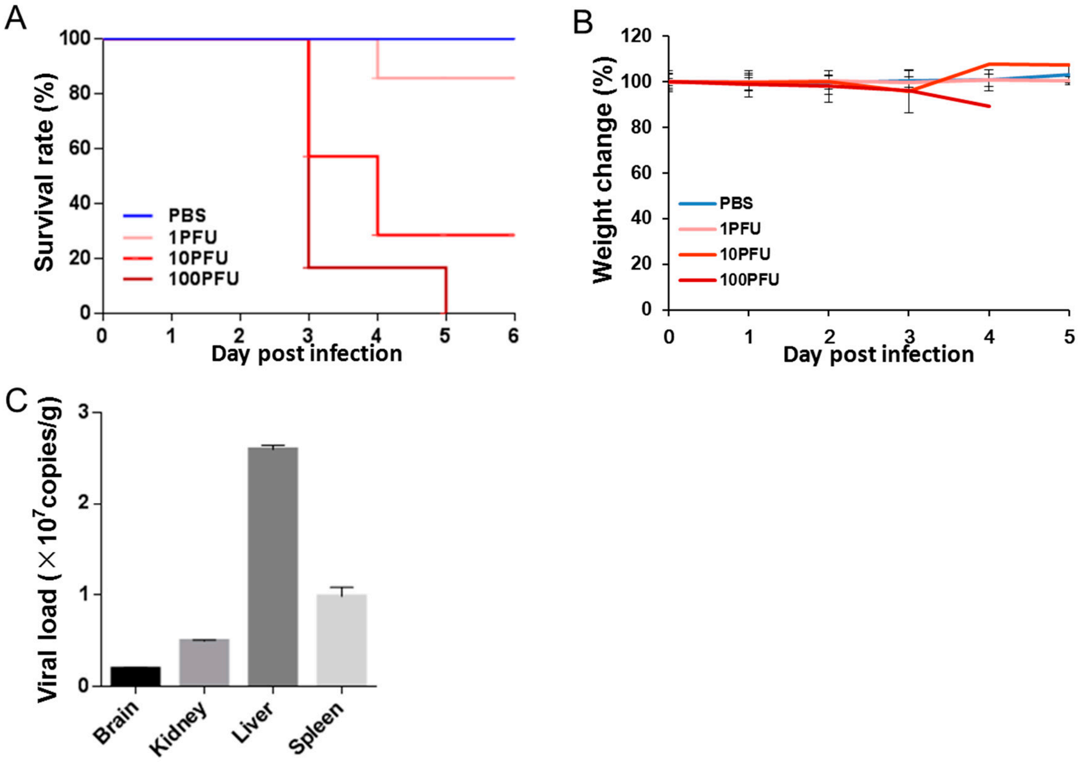

Figure. S3. Determination of LD_{50} for the rWT virus. 6-week-old BALB/c mice were intraperitoneally inoculated with 10-fold dilutions of rWT (ranging from 1 PFU to 100 PFU) or inoculated with PBS as a control. The mice in each group (n=6) were observed for 5 days after infection on a daily basis. The mice survival rates (A) and body weights (B) were recorded. Error bars represent standard deviation. (C) Tissue tropism of rWT. Brain, kidney, liver and spleen samples were collected from infected mice at 3 days post infection. RNA was extracted from tissue homogenates by using Trizol Reagent. RNA viral loads in brain, kidney, liver and spleen were detected by qRT-PCR.

Fig. S4.

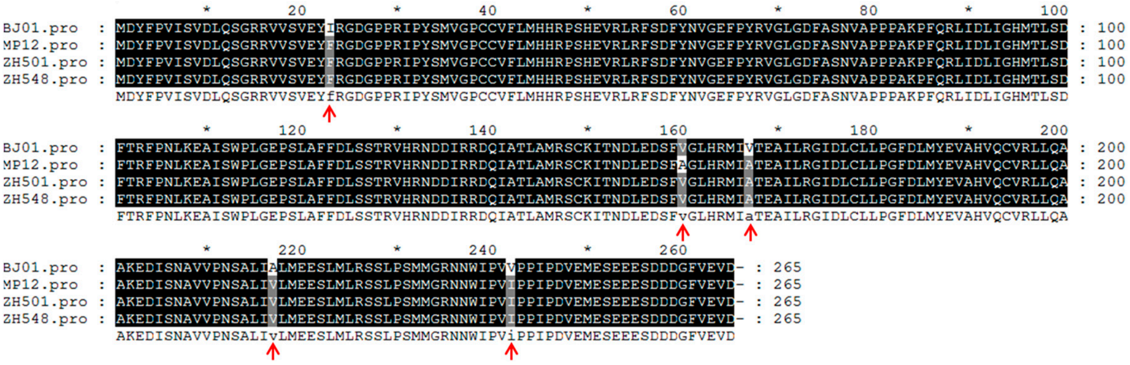

Figure. S4. Alignment of NSs amino acid sequences of BJ01, MP12, ZH501 and ZH548. Red arrows indicate the different amino acids between BJ01 and MP12 NSs proteins.
